# Controversies and conundrums in global surgery

**DOI:** 10.1093/bjs/znaf206

**Published:** 2025-12-10

**Authors:** Rachel Hargest, Rocco Friebel

**Affiliations:** Global Surgery Policy Unit, Royal College of Surgeons of England, London, UK; Cardiff University, Cardiff, UK; Global Surgery Policy Unit, Department of Health Policy, London School of Economics and Political Science, London, UK

In 2015, the Lancet Commission on Global Surgery reported the scale of the need for surgery in low- and middle-income countries (LMICs): 5 billion people lacking access to safe, affordable, and timely surgery; 33 million people facing catastrophic health expenditure each year; and a requirement for 143 million additional surgical procedures annually to deal with recognized surgical burden of disease^1^. Given the geopolitical, economic, and epidemiological events that have unfolded in the decade since the publication of this report, it is possible that these figures may be even worse now, as countries, which at the time had reasonably functioning health systems, have become embroiled in conflicts, beset by natural disasters, or experienced squeezes to their healthcare budgets. In a recent study we estimated surgical conditions alone to account for over 17 million deaths and 619 million disability adjusted life years, annually^2^. The need for surgeons to engage globally has never been more pressing.

There has been an exponential increase in interest in global surgery over the last two decades. This is evidenced by a significant rise in publications on global surgery topics^3^, the proliferation of educational courses in global surgery, and the enthusiasm of many doctors and medical students in high-income countries (HICs) for undertaking short-term charitable or humanitarian surgical missions, boot camps and other endeavours in LMICs. There is a general belief that such efforts are ‘a good thing’ and improve the clinical services and training opportunities for colleagues in LMICs, as well as engaging surgeons in more privileged parts of the world to devote their time and expertise to potentially worthwhile projects.

However, in recent years, it has also become apparent that there are some unintended consequences of global surgery efforts, which do little to improve surgical services in the recipient countries and may in fact be counterproductive^4^. For example, many surgical missions are short term and leave no sustainable legacy and may disrupt ongoing local services. Indeed, the humanitarian response to disasters has at times been poorly coordinated, inappropriate, and a source of shame on the medical profession^5^. Several of the papers in this issue address some of the unintended consequences of this increased interest in global surgery. Alayande and Bekele, from Rwanda, review progress against the Lancet Commission targets, Roy and international colleagues tackle the thorny topic of neocolonialism in global surgery, and Edge and Buccimazza from South Africa discuss the ethics of conducting clinical research in LMICs.

Although many individual surgeons are involved in humanitarian or other global surgery efforts voluntarily, these missions are not without cost. The scope and duration of missions are usually determined by the outgoing team and may or may not be an appropriate response to the needs of the recipient region or institution. The cost of arranging missions is significant, including transportation and insurance, and they rarely lead to capacity building or a sustainable service that exceeds the time horizon of the mission. It would be prudent to ask whether such funds would be better directed to investing in local surgical services. In this issue, the paper by Ifeanyichi and colleagues seeks to address this question by synthesizing and critically evaluating the available evidence on the cost of missions *versus* domestic investments in surgical care.

When considering training of surgical practitioners in LMICs, there are universities, colleges, and other professional associations running courses, summer schools, and boot camps in LMICs. However, many of these events are courses designed by and for surgeons practising in HICs, which may or may not be appropriate for transfer to LMIC circumstances. It is rare for surgeons practising in LMICs or conflict and disaster zones to be asked what, if any, education they need from an incoming team. The article by McKnight and colleagues in this issue describes the use of a training needs analysis (TNA) tool to determine the learning requirements of surgical and anaesthetic staff in Somaliland and the relevance of the skill sets taught to the job role of the individual healthcare worker.

In the world of academia, there is little evidence that collaborative research efforts are building capacity within LMIC universities and other professional organizations. In a recent review, we screened over 117 000 papers on global surgery and showed that the majority emanated from institutions in just six countries—USA, India, Brazil, China, South Africa, and the United Kingdom^3^. Moreover, first (42%) and last (41%) authors were disproportionately based in HICs and the topics for research were predominantly determined from the HIC collaborators or funders. There are many reasons why surgeons in LMICs are unable to undertake and publish research, one of which is the overwhelming clinical demands caused by conflict and other disasters. In this issue, Marks and colleagues report the experience of surgeons in conflict regions in the Middle East and North Africa, giving voice to the experience and perspective of surgeons who strive to provide safe, quality surgery in the most challenging of environments.

Funding of global surgery is another area of potential controversy^6^. Although many global health and global surgery programmes rely on philanthropic support, concerns have been raised about so called ‘philanthrocapitalism’^7,8^. This refers to individuals who have become extremely wealthy in capitalist systems, who then apply their skills and similar techniques that they used to create their wealth, to their efforts to give away their fortunes to particular projects. In May 2024, the World Health Organization (WHO) published a list of its donors for 2023, which shows that after the USA, the next largest donor to WHO is the Gates Foundation, ahead of China and smaller contributions from other governmental donors^9^. The extent to which such powerful philanthropic funders influence global health priorities has been questioned in recent years^10^. With the pronouncements from President Trump, the USA contribution to WHO funding is likely to decrease significantly leading to greater reliance on philanthrocapitalists and the contributions of China and other countries. It is possible that these changes will lead to a substantial realignment of global health priorities, impacting the work that will be undertaken by academia and non-governmental organizations. Importantly, it is likely that governments will have to act even more strategically, leveraging policies that are able to achieve incremental improvements in service delivery with fewer financial resources. This could include adopting task-shifting as a broader policy approach to develop surgical capacity^11^—a topic addressed in this issue through a political economy analysis by Kebede and colleagues.

In 2008 Farmer and Kim famously described surgery as the ‘neglected stepchild of global health’^12^. The focus of much global health funding has been on control of infectious diseases, vaccination and maternal and child health. Although these are all important global health issues, the financial case for investing in surgical services in LMICs has been recognized by the World Bank and other major funders, but has yet to be widely accepted within the Universal Health Coverage (UHC) packages currently being developed by LMICs. This comes despite a growing body of evidence showing that surgical interventions are highly cost-effective and an economically smart investment^13^. In this issue, Wharton and colleagues discuss the issues around funding of surgical services within UHC packages and the financial mechanisms which may underpin sustainable funding.

We hope that this series of Controversies and Conundrums in Global Surgery will stimulate *BJS* readers to consider the complexities and unintended consequences of well-intentioned global surgery activities. As surgeons, we all wish to see provision of safe, affordable surgical care to all who need our services, and as a profession, we should use our collective expertise to do this in the most ethical, efficient, and sustainable manner.
